# Clopidogrel Versus Aspirin as Monotherapy Following Dual Antiplatelet Therapy in Patients With Acute Coronary Syndrome Receiving a Drug‐Eluting Stent: A Systematic Literature Review and Meta‐Analysis

**DOI:** 10.1002/clc.24326

**Published:** 2024-08-29

**Authors:** Dirk Sibbing, Johny Nicolas, Alessandro Spirito, Birgit Vogel, Davide Cao, Wanda Stipek, Ellen Kasireddy, Andi Qian, Irfan Khan, Roxana Mehran

**Affiliations:** ^1^ Department of Internal Medicine Ludwig‐Maximilians‐Universität München Munich Germany; ^2^ Division of Cardiology Icahn School of Medicine at Mount Sinai New York New York USA; ^3^ Sanofi Bridgewater New Jersey USA; ^4^ Evidinno Research Outcomes Inc. Vancouver British Columbia Canada

**Keywords:** acute coronary syndrome, aspirin, clopidogrel, dual antiplatelet therapy, meta‐analysis, single antiplatelet therapy, systematic review

## Abstract

**Objective:**

This study aimed to evaluate the comparative effectiveness and safety of clopidogrel versus aspirin as monotherapy following adequate dual antiplatelet therapy (DAPT) in patients with acute coronary syndrome (ACS).

**Methods:**

MEDLINE, Embase, and CENTRAL were searched from database inception to September 1, 2023. Randomized controlled trials (RCTs) and observational studies evaluating the effectiveness or safety of clopidogrel versus aspirin as monotherapy following DAPT in patients with ACS who received a drug‐eluting stent were included. Random‐effects meta‐analyses were conducted to compare risks of major adverse cardiovascular events (MACE) and clinically relevant bleeding.

**Results:**

Of 6242 abstracts identified, three unique studies were included: one RCT and two retrospective cohort studies. Studies included a total of 7081 post‐percutaneous coronary intervention ACS patients, 4260 of whom received aspirin monotherapy and 2821 received clopidogrel monotherapy. Studies included variable proportions of patients with ST‐elevation myocardial infarction (STEMI), non‐STEMI, and unstable angina. From the meta‐analysis, clopidogrel was associated with a 28% reduction in the risk of MACE compared with aspirin (hazard ratio [HR]: 0.72; 95% confidence interval [CI]: 0.54, 0.98), with no significant difference in clinically relevant bleeding (HR: 0.92; 95% CI: 0.68, 1.24).

**Conclusion:**

Despite the paucity of published evidence on the effectiveness and safety of clopidogrel versus aspirin in patients with ACS post‐drug‐eluting stent implantation, this meta‐analysis suggests that clopidogrel versus aspirin may result in a lower risk of MACE, with a similar risk of major bleeding. The present results are hypothesis‐generating and further large RCTs comparing antiplatelet monotherapy options in ACS patients are warranted.

## Graphical abstract

Clopidogrel reduced the risk of major adverse cardiovascular events by 28% versus aspirin and there was a similar risk of major bleeding after adequate dual antiplatelet therapy in acute coronary syndrome patients post‐drug‐eluting stent implantation.

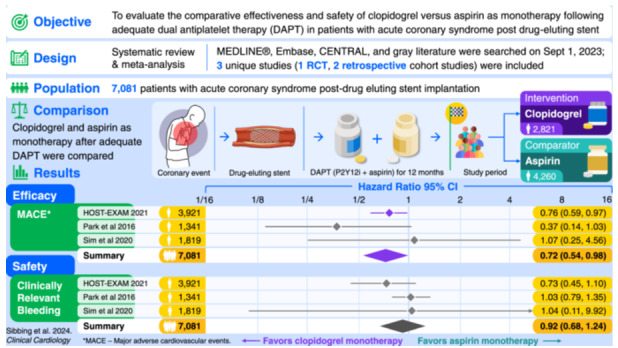

## 1 Introduction

Despite significant advances and improvements in revascularization in recent years, acute coronary syndrome (ACS) still remains associated with high rates of morbidity and mortality both globally and in the United States [1, 2]. Advances in the design of drug‐eluting stents (DESs) in particular have helped to improve outcomes in ACS; these have resulted from improved understanding of stent structure and composition, including polymer coating and antiproliferative agents, to suppress immune‐mediated hypersensitivity reactions that can cause stent thrombosis or late restenosis [3]. In ACS patients who undergo percutaneous intervention (PCI) using DES, dual antiplatelet therapy (DAPT; aspirin plus a P2Y12 inhibitor [P2Y12i]) is indicated before PCI, with subsequent continuation as a maintenance dose for at least 12 months post‐PCI to reduce the risk of early stent thrombosis and recurrent ischemic events [1, 4, 5]. However, many recent large studies have highlighted the benefits of shorter duration DAPT (1–3 months). These studies were included in recent United States and European guideline recommendations identifying cases and patient groups where shorter duration DAPT may, for example, reduce the risk of bleeding associated with longer courses [1, 5].

Following DAPT, antiplatelet monotherapy is recommended, with aspirin traditionally being the preferred option unless the patient has a contraindication or intolerance [6]. The European Society of Cardiology (ESC) published guidelines in 2023 recommending that single antiplatelet therapy, preferably with a P2Y12i, should be considered for patients who are event‐free after 3–6 months of DAPT and who are not high ischemic risk. Aspirin or P2Y12i monotherapy after 1 month of DAPT may also be considered in patients with high bleeding risk. The 2023 ESC guidelines also stated that P2Y12i may be considered as an alternative to aspirin for long‐term monotherapy; this recommendation represents a change from the 2020 Guidelines [1, 7]. Evidence to support long‐term clopidogrel monotherapy dates back to the CAPRIE trial in which clopidogrel was more efficacious than aspirin in reducing major adverse cardiovascular events (MACE) while reducing bleeding in patients with atherosclerotic cardiovascular disease and was also associated with a significantly lower rate of gastrointestinal hemorrhage events [8].

While the CAPRIE trial showed that clopidogrel was more effective than aspirin as monotherapy in reducing the combined risk of ischemic stroke, myocardial infarction (MI), or vascular death in patients at risk of ischemic events, it should be noted that this study also included non‐coronary patients and was conducted before the introduction of DES; therefore, it was not a post‐PCI study [8]. A recent meta‐analysis comparing P2Y12i versus aspirin as monotherapy for long‐term prevention of cardiovascular events also concluded that P2Y12i showed superior efficacy and similar overall safety; however, the analysis included patients with stable coronary artery disease and was therefore not focused on the ACS population post‐DES [7].

While there have been recent meta‐analyses comparing P2Y12i and aspirin for long‐term monotherapy in cardiovascular patients, there remains a paucity of evidence specific to the ACS population with DES implantation. Therefore, the aim of this systematic literature review and meta‐analysis was to evaluate the comparative effectiveness and safety of clopidogrel, a P2Y12i, versus aspirin as monotherapy following adequate DAPT in patients with ACS post‐DES implantation.

## 2 Methods

Standard methodologies for conducting and reporting systematic reviews as recommended by the Cochrane Handbook for Systematic Reviews of Interventions were followed [9]. Results for this review were reported according to the Preferred Reporting Items for Systematic Reviews and Meta‐Analyses (PRISMA) guidelines [10].

### 2.1 Data Sources and Search Strategies

Searches were conducted in MEDLINE, Embase, and CENTRAL (Cochrane Central Register of Controlled Trials) via OvidSP to capture records published up to September 1, 2023 (Supporting Information S1: Tables S1–S5). Additionally, conference abstracts published between 2021 and 2023 from the American College of Cardiology, the American Heart Association, the British Cardiovascular Society, the ESC, World Stroke, Transcatheter Cardiovascular Therapeutic, and EuroPCR congresses were searched. Searches of United States (http://clinicaltrials.gov) and European (https://www.clinicaltrialsregister.eu/) clinical trial registry databases were also conducted to find trials that had reported results that were not already published in peer‐reviewed journals. Lastly, “hand searches” of the reference lists of previously published literature reviews on the same topic were also conducted to capture additional eligible studies that were missed in the electronic database search.

### 2.2 Study Selection

Study eligibility criteria for the systematic review were defined using the Population, Intervention, Comparator, Outcome (PICO) framework. Randomized controlled trials (RCTs) and observational studies that evaluated the effectiveness or safety of clopidogrel versus aspirin as monotherapy following any duration of DAPT in adults with ACS (including ST‐elevation MI [STEMI], non‐STEMI [NSTEMI], or unstable angina) post‐DES implantation were included. The effectiveness outcomes of interest were MACE (and its individual components of MI, ischemic stroke, or mortality), stent thrombosis, coronary revascularization, and hospitalization due to unstable angina; safety outcomes of interest were bleeding events (total, major, minor, or fatal), adverse events (AEs), treatment‐related AEs, serious AEs, study withdrawal, and drug discontinuation or treatment switching after 1 year of DAPT. Studies on children or adolescents or those including ACS patients who received non‐urgent DES implantation (received 1+ years after the ACS event) or who received other P2Y12i such as ticagrelor or prasugrel were excluded. Non‐English publications were also excluded.

Two independent reviewers were responsible for reviewing all abstracts according to the PICO criteria. Abstracts considered eligible for inclusion proceeded to a full‐text screening phase, where they were screened in duplicate by the same reviewers. All records deemed eligible after full‐text screening were included in the review. At each stage of the screening process, any discrepancies between reviewers in the decision to include or exclude an article were resolved by a third reviewer to reach a consensus.

### 2.3 Data Extraction and Quality Assessment

Relevant data available from the publications were extracted independently by two reviewers, and if discrepancies in interpretation could not be resolved, a third reviewer was consulted to reach consensus. Data extraction included study characteristics, interventions, patient characteristics, and the safety and effectiveness outcomes of interest. Baseline patient characteristics of interest were age, sex, comorbidities, and type of ACS. In cases where outcome data of interest were not available in the publication, the study investigators were contacted to collect additional information. Specifically, information on the number and proportion of ACS patients who experienced Bleeding Academic Research Consortium (BARC) 3/5 bleeding in the HOST‐EXAM trial at 24 months was kindly provided by the clinical trial investigators.

Two independent reviewers assessed the quality of the included studies using the Cochrane Risk of Bias Tool (RoB 2) for randomized trials [11] and the Newcastle–Ottawa Scale for non‐randomized studies [12]. Following reconciliation between the decisions of the two reviewers, a third reviewer intervened to reach consensus if there were any remaining unresolved conflicts.

### 2.4 Statistical Analysis

Before the meta‐analysis, a full feasibility assessment was completed [13] to determine whether a quantitative analysis could be conducted. Following this assessment, two analyses were conducted to study the effectiveness and safety of clopidogrel versus aspirin as monotherapy. The effectiveness analysis was a pairwise meta‐analysis on MACE, while the safety analysis was a pairwise meta‐analysis on clinically relevant bleeding, defined using either the BARC 3/5 or thrombolysis in myocardial infarction (TIMI) major bleeding criteria. Random‐effects meta‐analyses were conducted using the metafor R package. Inverse‐variance weights were used to calculate the pooled relative treatment effect.

## 3 Results

### 3.1 Study Selection

**Figure 1:**
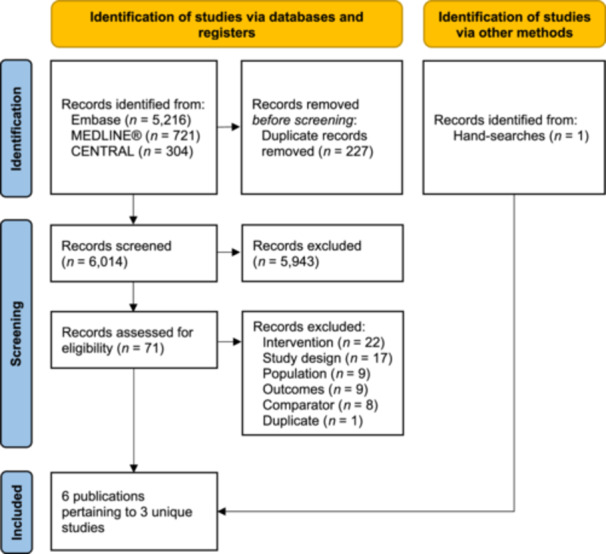
PRISMA flow diagram. *n*, records.

In total, 6241 records were identified from the review via MEDLINE, Embase, and CENTRAL (Figure 1); one additional record was included from hand searching. After full‐text screening, a total of three unique studies pertaining to six publications were included in the review and meta‐analysis.

### 3.2 Study Characteristics

Of the three primary studies included, one was an RCT and two were observational retrospective cohort studies (Table 1). Sample sizes of ACS patients were 3921 in the HOST‐EXAM trial [14], and 1341 and 1819 in the observational studies by Park et al. [15] and Sim et al. [16], respectively. All studies were conducted in South Korea. It is noteworthy that four publications pertaining to the HOST‐EXAM trial were included in the review, the original publication [14], a study extension [17], a conference abstract [18], and a letter to the editor [17] reporting the results of a subgroup analysis comparing outcomes for ACS versus non‐ACS patients. The definitions of MACE in the three primary studies were broadly similar, with the following caveats noted: all‐cause death was a component of the MACE composite endpoint in HOST‐EXAM and Sim et al. [16], whereas cardiac death only was included in Park et al. [15]; stroke was included in the composite endpoint in Park et al. [15] and HOST‐EXAM, whereas in Sim et al. [16], ischemic stroke was included; repeat PCI and stent thrombosis were included in Sim et al. [16]; and readmission due to ACS and major bleeding events (BARC type ≥ 3) were included in Park et al. [15].

**Table 1:** Study characteristics across included studies.

Study	Study design	Study country	Intervention	*N*	Definition of MACE
HOST‐EXAM [14, 17, 18]	RCT, multicenter, open‐label, Ph IV	South Korea	Clopidogrel	1964	Composite of all‐cause death, nonfatal myocardial infarction, stroke, readmission due to ACS, and major bleeding events (BARC type ≥ 3)
			Aspirin	1957	
Park et al. [15]	Observational, retrospective cohort, single‐center	South Korea	Clopidogrel	324	Composite of cardiac death, myocardial infarction, or stroke; all deaths were considered cardiac‐related unless a definite noncardiac cause could be established
			Aspirin	1017	
Sim et al. [16]	Observational, retrospective cohort, multicenter	South Korea	Clopidogrel	533^a^	Composite of death from any cause, MI, repeat PCI, stent thrombosis, or ischemic stroke^b^
			Aspirin	1286^a^	

Abbreviations: ACS, acute coronary syndrome; BARC, Bleeding Academic Research Consortium; MACE, major adverse cardiovascular events; MI, myocardial infarction; PCI, percutaneous intervention; RCT, Randomized controlled trial.

^a^ Number of patients corresponds to the population analyzed in the inverse probability of treatment‐weighted analysis.

^b^ Outcome was reported as major adverse cardiovascular and cerebrovascular events (MACCE).

Intervention and baseline patient characteristics are summarized in Supporting Information S1: Table S6. Baseline characteristics in ACS patients, including age, sex, mean left ventricular ejection fraction (LVEF), medical history, comorbidities, and type of stent, were reported only by Sim et al. [16]. Within the respective aspirin and clopidogrel groups in this study, the mean age was 60.7 and 62.2 years, the proportion of female participants was 19.8% and 26.0%, and the mean LVEF was 53.1% and 53.4%, respectively [16]. History of MI, ischemic stroke, hemorrhagic stroke, or heart failure were present in fewer than 4% of patients. Smoking, hypertension, and diabetes were common characteristics, occurring in roughly 60%, 50%, and 20%, respectively, in the study population. Patients received either an everolimus‐eluting, zotarolimus‐eluting, biolimus‐eluting, sirolimus‐eluting, or other type of stent, with the everolimus‐eluting stent being the most frequently used stent among patients (43.3%–45.3%). Detailed baseline patient characteristics for HOST‐EXAM and Park et al. [15] were only reported for their primary populations, which included a mix of ACS and non‐ACS (stable angina) patients. Therefore, patient characteristics were not available for the ACS subgroups in these studies. Additionally, it should be noted that for both observational studies by Sim et al. [16] and Park et al. [15], authors utilized an inverse probability of treatment weighting approach based on propensity scores to control for differences in baseline characteristics and potential confounding factors. Covariates for multiple logistic regression analyses included relevant clinical variables such as age, sex, comorbidities (e.g., diabetes mellitus), history of MI, type of DES, and DAPT duration, among others.

**Figure 2:**
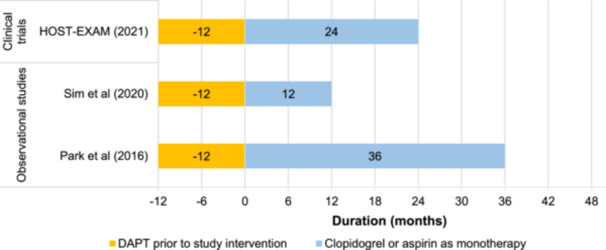
Duration of DAPT and subsequent clopidogrel or aspirin as monotherapy across included studies. DAPT, dual antiplatelet therapy. *Note:* The start of follow‐up was considered to be at timepoint 0. The HOST‐EXAM extension trial reported a treatment duration of a median of 5.8 years for antiplatelet monotherapy.

Both observational studies reported a treatment duration of 12 months for DAPT following DES implantation before switching to monotherapy. In HOST‐EXAM, the duration of DAPT was 6–18 months (i.e., 12 ± 6 months; Figure 2). The median time from randomization to monotherapy in HOST‐EXAM was 382 days [14]. In all three studies, included patients were event‐free throughout DAPT, and patients who experienced MACE, repeat revascularization, or major bleeding were excluded from the subsequent monotherapy comparison.

The included studies were more heterogeneous in terms of treatment duration and follow‐up for monotherapy following DAPT (Figure 2). The duration of clopidogrel or aspirin as monotherapy ranged from 12 [16] to 36 [15] months. It is noteworthy that the HOST‐EXAM extension trial reported a treatment duration of a median of 5.8 years for monotherapy [17]. Clopidogrel loading dose was only reported in the HOST‐EXAM trial. In patients who previously received DAPT with ticagrelor, the subsequent clopidogrel monotherapy loading dose was 600 mg, and previous prasugrel DAPT users randomized to subsequent clopidogrel monotherapy switched to clopidogrel maintenance therapy without a loading dose [14]. Following DAPT, the maintenance dose of clopidogrel was administered as 75 mg once daily in HOST‐EXAM and Sim et al. [16]. Aspirin monotherapy was administered at a dose of 100 mg once daily in these same two studies. Park et al. [15] did not report dosing information.

**Figure 3:**
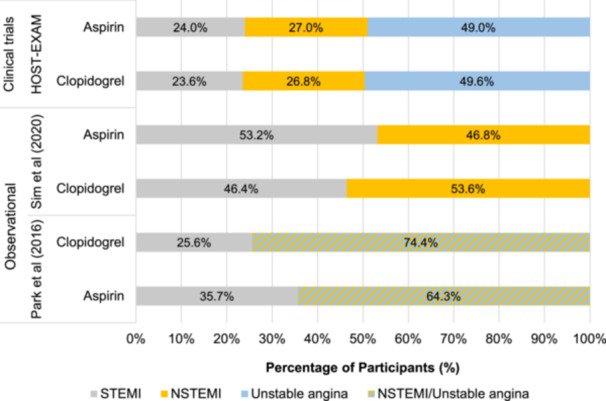
Distribution of the type of acute coronary syndrome across studies. NSTEMI, non‐ST‐elevation myocardial infarction; STEMI, ST‐elevation myocardial infarction.

With respect to MI subtypes, proportions of patients presenting with STEMI and NSTEMI ranged from 23.6% [14] to 53.2% [16] and from 26.8% [14] to 53.6% [16], respectively (Figure 3). Unstable angina was reported by the HOST‐EXAM trial and Park et al. [15]. Park et al. reported the percentage of patients with unstable angina and NSTEMI grouped together. Across the two treatment arms in this study, the percentages of patients with unstable angina or NSTEMI were 64.3% and 74.4%. As per the PICO criteria, all patients were treated with a DES.

### 3.3 Study Quality Assessment and Risk of Bias

The Cochrane RoB 2 tool for randomized trials and the Newcastle–Ottawa scale for observational studies were used for study quality assessment (Supporting Information S1: Tables S7 and S8). In summary, the included studies were of generally high or moderate quality, indicated by some concerns in the Cochrane RoB 2 tool (due to the lack of blinding of the participants and study personnel to the treatment being administered), and total scores of at least 8 out of a possible 9 points for the Newcastle–Ottawa scale.

### 3.4 Meta‐Analysis

All three primary studies were included in the meta‐analysis for effectiveness and safety. It is noteworthy that data from the HOST‐EXAM extension study [17] were not used in the meta‐analysis as the median follow‐up (5.8 years) was too dissimilar to the time points reported by the other studies.

#### 3.4.1 Effectiveness Analysis

**Figure 4:**
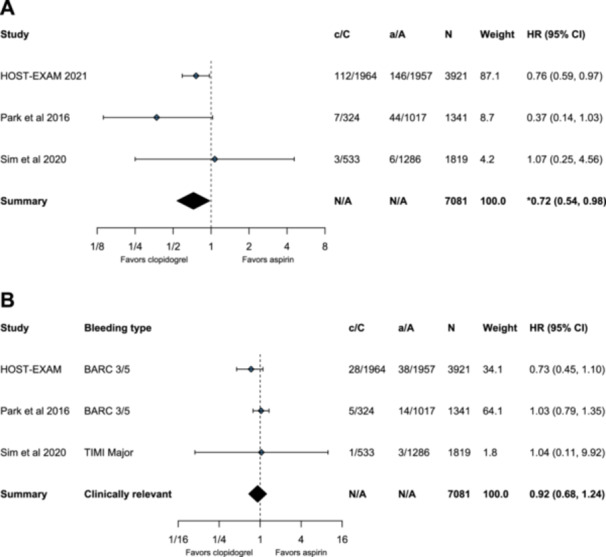
Forests plot of (A) major adverse cardiovascular events and (B) clinically relevant bleeding for clopidogrel versus aspirin as monotherapy. * Denotes statistical significance. a, number of events in the aspirin arm; A, number of aspirin‐treated patients in the study; c, number of events in the clopidogrel arm; C, number of clopidogrel‐treated patients in the study; CI, confidence interval; HR, hazard ratio; N, number of patients; N/A, not applicable; Weight, inverse‐variance weights.

Only one of the included studies reported a statistically significant within‐study difference in MACE between treatments, with a significantly lower incidence of MACE for patients treated with clopidogrel compared with aspirin as monotherapy at 24 months [14]. The other two studies reported no significant difference in MACE between treatments [15, 16]. In the meta‐analysis, clopidogrel compared with aspirin as monotherapy resulted in a 28% reduction in the risk of MACE (hazard ratio [HR]: 0.72; 95% confidence interval [CI]: 0.54, 0.98; Figure 4A).

#### 3.4.2 Safety Analysis

None of the included studies reported a statistically significant difference in clinically relevant bleeding between treatments in ACS patients [15, 16, 18]. In the meta‐analysis, there was no significant difference between clopidogrel and aspirin as monotherapy for clinically relevant bleeding (HR: 0.92; 95% CI: 0.68, 1.24; Figure 4B).

### 3.5 Other Outcomes

Sim et al. [16] was the only study to report the percentage of patients who experienced stent thrombosis or coronary revascularization in the ACS population. Twelve months after initiation of the study intervention, 0.2% of patients receiving clopidogrel had stent thrombosis, while this was 0% for patients receiving aspirin (*p* = 0.121). Of patients receiving clopidogrel, the proportions of patients undergoing target and non‐target vessel revascularization were 0% and 0.2% (*p* = 0.362), respectively. These proportions were 0.2% and 0.1% (*p* = 0.535) for patients receiving aspirin.

Other outcomes of interest included hospitalization due to unstable angina, fatal bleeding, AEs, treatment‐related AEs, serious AEs, study withdrawal, and drug discontinuation or treatment switching after 1 year of DAPT; however, no studies reported these outcomes for the specific population of interest.

## 4 Discussion

The aim of this systematic review and meta‐analysis was to describe and characterize the landscape of evidence on, as well as to quantify, the effectiveness and safety of clopidogrel versus aspirin as monotherapy after adequate DAPT therapy in patients with ACS post‐DES implantation. A small number of studies, all of which were multicenter studies conducted in South Korea, were identified. The included studies varied according to the follow‐up time, with 12, 24, and 36 months of follow‐up, and only one study reported a significant difference in MACE between treatments, favoring clopidogrel versus aspirin as monotherapy at 24 months [14]. This meta‐analysis of MACE indicated a statistically significant 28% reduction in risk (HR: 0.72; 95% CI: 0.54, 0.98) with clopidogrel versus aspirin as monotherapy. For safety, clinically relevant bleeding outcomes (defined as either BARC 3/5 bleeding or TIMI major bleeding) were available for all included studies. Both within‐study findings and the meta‐analysis results did not find a significant difference in clinically relevant bleeding between clopidogrel and aspirin as monotherapy. Notably, in the analysis of clinically relevant bleeding, Sim et al. [16] showed a wide CI, perhaps due to the low number of events in the study—with one event in the clopidogrel arm and three with aspirin. Additionally, the definition of TIMI major bleeding, which was only used in Sim et al. [16], is stricter than that of BARC 3/5 bleeding, which would conceivably yield fewer counted major bleeding events. Results from the HOST‐EXAM trial were also available from the ACS subgroup at a median follow‐up of 5.8 years [17]. Although the results were not included in the meta‐analysis to maintain time‐point homogeneity across included studies, there was evidence of a trend for reduced bleeding in the clopidogrel monotherapy arm, although it did not reach statistical significance in the ACS subgroup (HR: 0.85; 95% CI: 0.65, 1.13).

To the best of our knowledge, this is the first systematic review and meta‐analysis solely focusing on patients with ACS who received clopidogrel or aspirin as monotherapy following DAPT post‐DES. Previous meta‐analyses have investigated the effectiveness and safety of clopidogrel compared with aspirin as monotherapy following DAPT within the broader population of coronary artery disease patients with or without PCI [19, 20, 21]. The PANTHER meta‐analysis, conducted by Gragnano et al., was a patient‐level meta‐analysis of RCTs comparing P2Y12i versus aspirin as monotherapy for the prevention of cardiovascular events in patients with established coronary artery disease [19]. Patient‐level data were collected from seven RCTs, and of the patients who were grouped in the P2Y12i arm, 62.0% received clopidogrel. The risk of MACE was significantly lower with P2Y12i compared with aspirin over 2 years (HR: 0.88; 95% CI: 0.79, 0.97). Furthermore, the risk of major bleeding was similar between groups (HR: 0.87; 95% CI: 0.70, 1.09), and the treatment effects were noted to be consistent across types of P2Y12i. Tan et al. [21] conducted a systematic review and meta‐analysis of clinical trials and observational studies aimed at assessing the efficacy and safety of clopidogrel versus aspirin in the post‐PCI population after completion of DAPT. They identified a total of five relevant publications, including the HOST‐EXAM trial and the observational study by Park et al. [15]. The remaining studies in their evidence base reported data on mixed populations of ACS and non‐ACS patients (including stable angina) and were therefore not included in the current review. This meta‐analysis of post‐PCI patients including stable angina found consistent results with the present study; their findings also suggested that clopidogrel versus aspirin as monotherapy was associated with a significant reduction in MACE (risk ratio [RR]: 0.77; 95% CI: 0.65, 0.91), with no significant difference in major bleeding (RR: 0.74; 95% CI: 0.43, 1.29). Ando et al. conducted a systematic review and network meta‐analysis of RCTs to compare aspirin versus any P2Y12i after DAPT discontinuation in patients who underwent PCI [20]. Their review captured a total of 19 studies, including the HOST‐EXAM trial. The authors did not analyze MACE; however, the risk of their primary efficacy endpoint, MI, was significantly higher with aspirin compared with any P2Y12i (RR: 1.32; 95% CI: 1.08, 1.62). In their meta‐analysis, comparisons between either ticagrelor (RR: 0.69; 95% CI: 0.63, 0.90) or clopidogrel (RR: 0.74; 95% CI: 0.46, 1.19) versus aspirin as monotherapy showed reduced MI risk with the P2Y12i; however, results did not achieve statistical significance. An analysis of clopidogrel versus aspirin was not conducted for major bleeding, although no significant difference was seen between aspirin and monotherapy with any P2Y12i for the risk of major bleeding (RR: 1.12; 95% CI: 0.82, 1.53). The findings reported in these prior studies are consistent with the current meta‐analysis.

Notably, there is recent and increasing evidence emerging on the use of alternative strategies in select patients undergoing PCI, such as short DAPT or DAPT de‐escalation to P2Y12i monotherapy. For example, a recent systematic review and network meta‐analysis of 23 RCTs by De Filippo et al. [22] investigated six different antiplatelet therapy strategies including de‐escalation in patients undergoing PCI [22]. Results were largely inconclusive, given varying levels of uncertainty; however, this may highlight a need for further investigation within subpopulations of patients. Indeed, recent guidelines have recognized the reduced bleeding benefits associated with alternative strategies, such as use of the shorter DAPT in patients who are event‐free after 3 to 6 months and who are not at high risk of ischemic events [1]. Further, it is recommended that in high‐bleeding risk patients, aspirin or a P2Y12i as monotherapy may be considered as early as after 1 month of DAPT with a potent P2Y12i. In light of this, there appears to be a growing movement to explore alternative and personalized strategies in this population. However, much of the existing literature on this topic is still limited to studies focused on carefully selected patient populations. Upon the emergence of new evidence on clopidogrel versus aspirin as monotherapy following DAPT in ACS patients post‐PCI, subgroup analyses on special or high‐risk populations are warranted to determine optimal strategies for individual patients. Furthermore, DAPT modulation strategies targeting the early period following PCI in ACS patients warrant specific assessment in dedicated studies and meta‐analyses.

The present review had several strengths. First, all stages of the review were carried out in accordance with standard recommendations for the conduct of systematic reviews [9, 10]. Second, the literature search and screening were comprehensive. Three major electronic databases and conference proceedings were covered during the searches and study selection. The included studies were generally of good quality or showed a low risk of bias. This is also the first review solely focusing on patients with ACS who received clopidogrel SAPT following DAPT post‐DES. Further, studies used broadly similar definitions of MACE, resulting in homogeneity in outcome definition, which is favorable in the context of a meta‐analysis. Other strengths include similarities in DAPT duration (~12 months) and adequate follow‐up duration of monotherapy treatment across studies.

Several limitations of this research should also be considered. The most important perhaps concerns the recognized paucity of available evidence. Indeed, as one of the strengths is the focus on post‐PCI patients with ACS, the same focus inherently limits the included studies. An important outcome was major bleeding (BARC 3/5 or TIMI major), and therefore, clinically relevant bleeding of lesser severity was not captured. Lastly, although we did not include a geographical restriction within our study inclusion criteria, all included studies were conducted in South Korea. This may limit the generalizability of the current findings. The paucity of evidence highlights an evidence gap in ACS patients, especially from the perspective of data from large, multinational RCTs including patients who underwent PCI with the latest generation DES. Future research providing patient‐level data in RCTs with more international institutions and clear delineation between ACS and non‐ACS patients would help to clarify the important research questions concerning this large patient population.

## 5 Conclusions

This systematic review identified a small number of studies evaluating the effectiveness or safety of clopidogrel versus aspirin as monotherapy following adequate DAPT in patients with ACS post‐DES implantation. Also, all studies identified were conducted within South Korea, which may limit the generalizability of the present findings. There is thus a paucity of published evidence for this important question. Nevertheless, a meta‐analysis of the currently available evidence suggests that clopidogrel may result in a lower risk of MACE, with a similar risk of clinically relevant bleeding compared with aspirin. While the evidence in this specific population (ACS with DES) is limited, the current results were consistent with findings from studies with broader populations (coronary artery disease or PCI including chronic coronary syndrome).

The results of this meta‐analysis are hypothesis‐generating, and further large RCTs comparing monotherapy options following adequate DAPT within this specific population of ACS with DES are required.

## Conflicts of Interest

Dirk Sibbing reports consulting fees and payment or honoraria for lectures, presentations, speakers bureaus, manuscript writing, or educational events from Bayer, Daiichi Sankyo, and Sanofi, outside of the present work. Alessandro Spirito reports a research Grant from the Swiss National Science Foundation, outside of the present study. Davide Cao reports honoraria for lectures, presentations, speakers bureaus, manuscript writing, or educational events from Terumo. Wanda Stipek and Irfan Khan are employees and shareholders at Sanofi (Bridgewater, NJ). Ellen Kasireddy and Andi Qian are employed by Evidinno Outcomes Research Inc. (Vancouver, BC, Canada), which was contracted by Sanofi to conduct this study. Roxana Mehran has received grants for institutional research from Abbott, Abiomed, Affluent Medical, Alleviant Medical, Amgen, AM‐Pharma, Arena Pharmaceuticals, AstraZeneca, Biosensors, Biotronik, Boston Scientific, Bristol Myers Squibb, CardiaWave, CeloNova, Chiesi, Cleerly Health Inc., Concept Medical, CSL Behring, Cytosorbents, Daiichi Sankyo, Element Science, Faraday Pharmaceuticals, Humacyte, Idorsia Pharmaceuticals, Janssen, Mediasphere Medical, Medtelligence, Medtronic, Novartis, OrbusNeich, Penumbra, PhaseBio, Philips, Pi‐Cardia, PLx Pharma, Protembis, ReCore Medical Inc., RenalPro, RM Global, Sanofi, Shockwave, Transverse Medical, Vivasure, and Zoll Medical. She has received consulting fees from Affluent Medical, Cordis, Henry Ford Health Cardiology, Novartis, Boehringer Ingelheim‐Lilly Partners, Ionis Pharamaceuticals, MedCon International, Novo Nordisk, Peerview Institute for Medical Education, TERUMO Europe N.V., Vectura Inc., VoxMedica, IQVIA, Radcliffe, TARSUS Cardiology, and WebMD. She is an StC Member of the Board of trustees of the American College of Cardiology, and she is an Associate Editor for JAMA. She is a member of the Scientific Advisory Board for AMA, a member of the American College of Cardiology BOT (SC Member CTR Program), and a member of the Women in Innovations Committee for the Society for Cardiovascular Angiography & Interventions. Roxana Mehran reports equity of less than 1% for Elixir Medical, Stel, and ControlRad. She is also a faculty member of the Cardiovascular Research Foundation (no fee). The remaining authors declare no conflict of interest.

## Supporting information

Supporting information.

## Data Availability

The data that support the findings of this study are available from the corresponding author upon reasonable request.
