# Supplementary material for: Clopidogrel Versus Aspirin as Monotherapy Following Dual Antiplatelet Therapy in Patients With Acute Coronary Syndrome Receiving a Drug‐Eluting Stent: A Systematic Literature Review and Meta‐Analysis
Source: Clin Cardiol. 2024 Aug 29;47(9):e24326. doi: 10.1002/clc.24326 (PMC11358762; doi:10.1002/clc.24326)
Supplement: Supplementary file 1 — Supporting information. [file CLC-47-e24326-s001.docx]

**Supplementary Material**

**Supplementary Table S1**: Search strategy for Embase via OvidSP

| **Database: Embase 1974 to present**  **Search executed: September 1, 2023** | | |
| --- | --- | --- |
| **#** | **String** | **Hits** |
| 1 | exp acute coronary syndrome/ | 75070 |
| 2 | exp myocardial infarction/ | 445647 |
| 3 | (("acute coronary" adj syndrome*) or "acs" or "Myocardial Infarction" or "AMI" or "STEMI" or "NSTEMI" or "Unstable Angina" or "Myocardial preinfarction syndrome" or "Preinfarction angina").ti,ab. | 391419 |
| 4 | or/1-3 | 565657 |
| 5 | exp drug eluting stent/ | 40453 |
| 6 | ((drug adj3 stent*) or DES).ti,ab. | 62440 |
| 7 | or/5-6 | 80802 |
| 8 | exp clopidogrel/ | 74609 |
| 9 | ("clopidogrel" or "SC 25989C" or "SC25989C" or "SC-25989C" or "SC 25990C" or "SC25990C" or "SC-25990C" or "SR 25989" or "SR25989" or "SR-25989" or "PCR 4099" or "PCR4099" or "PCR-4099" or iscover* or plavix* or duoplavin* or duocover* or co-plavix* or coplavix*).ti,ab. | 27975 |
| 10 | or/8-9 | 76657 |
| 11 | and/4,7,10 | 6797 |
| 12 | (conference paper or conference abstract).pt. | 5638109 |
| 13 | limit 12 to yr="2021-current" | 735308 |
| 14 | 12 not 13 | 4902801 |
| 15 | (exp animal/ or nonhuman/) not exp human/ | 7140549 |
| 16 | (book or chapter or editorial or erratum or letter or note or short survey or tombstone or comment or practice-guideline or journal correspondence or posters or news or newspaper article or lectures or interview or historical article or handbooks or guidelines or guidebooks or essays or database or catalogs).pt. | 3745866 |
| 17 | or/14-16 | 15048350 |
| 18 | 11 not 17 | 5336 |
| 19 | limit 18 to english language | 5216 |

**Supplementary Table S2**: Search strategy for MEDLINE® via OvidSP

| **Database: Ovid MEDLINE(R) ALL 1946 to present**  **Search executed: September 1, 2023** | | |
| --- | --- | --- |
| **#** | **String** | **Hits** |
| 1 | exp acute coronary syndrome/ | 20199 |
| 2 | exp myocardial infarction/ | 194216 |
| 3 | (("acute coronary" adj syndrome*) or "acs" or "Myocardial Infarction" or "AMI" or "STEMI" or "NSTEMI" or "Unstable Angina" or "Myocardial preinfarction syndrome" or "Preinfarction angina").ti,ab. | 253557 |
| 4 | or/1-3 | 324025 |
| 5 | exp drug-eluting stents/ | 13669 |
| 6 | ((drug adj3 stent*) or DES).ti,ab. | 70686 |
| 7 | or/5-6 | 76261 |
| 8 | exp clopidogrel/ | 10222 |
| 9 | ("clopidogrel" or "SC 25989C" or "SC25989C" or "SC-25989C" or "SC 25990C" or "SC25990C" or "SC-25990C" or "SR 25989" or "SR25989" or "SR-25989" or "PCR 4099" or "PCR4099" or "PCR-4099" or iscover* or plavix* or duoplavin* or duocover* or co-plavix* or coplavix*).ti,ab. | 14517 |
| 10 | or/8-9 | 16500 |
| 11 | and/4,7,10 | 826 |
| 12 | (exp animal/ or nonhuman/) not exp human/ | 5150978 |
| 13 | (book or chapter or editorial or erratum or letter or note or short survey or tombstone or comment or practice-guideline or journal correspondence or posters or news or newspaper article or lectures or interview or historical article or handbooks or guidelines or guidebooks or essays or database or catalogs).pt. | 2804808 |
| 14 | or/12-13 | 7860498 |
| 15 | 11 not 14 | 786 |
| 16 | limit 15 to english | 721 |

**Supplementary Table S3**: Search strategy for CENTRAL via OvidSP

| **Database: EBM Reviews - Cochrane Central Register of Controlled Trials July 2023**  **Search executed: September 1, 2023** | | |
| --- | --- | --- |
| **#** | **String** | **Hits** |
| 1 | exp acute coronary syndrome/ | 3274 |
| 2 | exp myocardial infarction/ | 13842 |
| 3 | (acute coronary syndrome$ or acs or Myocardial Infarction or AMI or STEMI or NSTEMI or Unstable Angina or Myocardial preinfarction syndrome or Preinfarction angina).ti,ab. | 38796 |
| 4 | or/1-3 | 40960 |
| 5 | exp Drug-Eluting Stents/ | 1909 |
| 6 | ((drug adj3 stent*) or DES).ti,ab. | 6160 |
| 7 | or/5-6 | 6891 |
| 8 | ("clopidogrel" or "SC 25989C" or "SC25989C" or "SC-25989C" or "SC 25990C" or "SC25990C" or "SC-25990C" or "SR 25989" or "SR25989" or "SR-25989" or "PCR 4099" or "PCR4099" or "PCR-4099" or iscover* or plavix* or duoplavin* or duocover* or co-plavix* or coplavix*).ti,ab. | 5209 |
| 9 | and/4,7-8 | 312 |
| 10 | limit 9 to english | 304 |

**Supplementary Table S4**: Search strategy for US clinical trial registry (https://clinicaltrials.gov/)

| **Database: ClinicalTrials.gov**  **Date of search: September 1, 2023** | | |
| --- | --- | --- |
| **#** | **Search String** | **Hits** |
| 1 | Condition or Disease: "acute coronary syndrome" OR "myocardial infarction" OR "AMI" OR "STEMI" OR "NSTEMI" OR "unstable angina" OR "myocardial preinfarction syndrome" OR "preinfarction angina"  Intervention/treatment: (“clopidogrel” OR "plavix") AND "aspirin"  Study Results: Studies With Results  Phase: 2-4 | 20 |

**Supplementary Table S5**: Search strategy for EU clinical trial registry (https://www.clinicaltrialsregister.eu/)

| **Database: ClinicalTrialsRegister.eu**  **Date of search: September 1, 2023** | | |
| --- | --- | --- |
| **#** | **Search String** | **Hits** |
| 1 | Search Terms: "acute coronary syndrome" OR "myocardial infarction" OR "AMI" OR "STEMI" OR "NSTEMI" OR "unstable angina" OR "myocardial preinfarction syndrome" OR "preinfarction angina") AND ("clopidogrel" OR "plavix") AND “aspirin”  Results Status: Trials with results | 49 |

**Supplementary Table S6**: Intervention and baseline patient characteristics for patients with acute coronary syndrome within the included studies

| **First Author & Year** | **Intervention** | **N** | **Mean Age (SD), Years** | **Female, n (%)** | **Type of ACS, n (%)** | **Medical History and Comorbidities, n (%)** | **Mean LVEF, % (SD)** | **Type of Stent Used, n (%)** |
| --- | --- | --- | --- | --- | --- | --- | --- | --- |
| HOST-EXAM (2021) | Aspirin 100 mg/day | 1957 | - | - | STEMI: 470 (24.0)  NSTEMI: 528 (27.0)  UA: 959 (49.0) | - | - | Any drug-eluting: 1957 (100), predominantly second generation drug-eluting stent ^a^ |
|  | Clopidogrel 75 mg/day | 1964 | - | - | STEMI: 463 (23.6)  NSTEMI: 526 (26.8)  UA: 975 (49.6) | - | - | Any drug-eluting: 1964 (100), predominantly second generation drug-eluting stent ^a^ |
| Park et al (2016) | Aspirin | 1017 | - | - | STEMI: 363 (35.7)  NSTEMI/UA: 654 (64.3) | - | - | Any drug-eluting: 1017 (100) |
|  | Clopidogrel | 324 | - | - | STEMI: 83 (25.6)  NSTEMI/UA: 241 (74.4) | - | - | Any drug-eluting: 324 (100) |
| Sim et al (2020) | Aspirin 100 mg/day | 1285 | 60.7 (11.7) | 255 (19.8) | STEMI: 684 (53.2)  NSTEMI: 601 (46.8) | Diabetes: 282 (22.0)  Dyslipidemia: 161 (12.5)  Hypertension: 574 (44.7)  History of MI: 37 (2.9)  History of ischemic stroke: 38 (3.0)  History of hemorrhagic stroke: 8 (0.6)  History of heart failure: 9 (0.7) | 53.40 (9.50) | Sirolimus-eluting: 25 (2.0)  Everolimus-eluting: 557 (43.3)  Zotarolimus-eluting: 396 (30.8)  Biolimus-eluting: 274 (21.3)  Other drug-eluting: 33 (2.6) |
|  | Clopidogrel 75 mg/day | 534 | 62.20 (11.7) | 139 (26.0) | STEMI: 248 (46.4)  NSTEMI: 286 (53.6) | Diabetes: 109 (20.4)  Dyslipidemia: 84 (15.7)  Hypertension: 260 (48.7)  History of MI: 16 (3.0)  History of ischemic stroke: 20 (3.8)  History of hemorrhagic stroke: 5 (0.9)  History of heart failure: 4 (0.8) | 53.10 (9.90) | Sirolimus-eluting: 20 (3.8)  Everolimus-eluting: 242 (45.3)  Zotarolimus-eluting: 135 (25.3)  Biolimus-eluting: 116 (21.7)  Other drug-eluting: 21 (3.9) |

ACS – Acute coronary syndrome; LVEF – Left ventricular ejection fraction; MI – Myocardial Infarction; NR – Not reported for a group or sub-group of patients with acute coronary syndrome; NSTEMI – Non-ST-elevation myocardial infarction; SD – Standard deviation; STEMI – ST-elevation myocardial infarction; UA – Unstable angina.

^a^ 97% of the primary population of the HOST-EXAM trial were treated with second generation drug-eluting stents, while remaining were treated with first-generation stents or unknown generation stents.

**Supplementary Table S7**: Summary of the Cochrane risk of bias 2 assessment for randomized trials

| **Trial name** | **Randomization process** | **Deviations from the intended interventions** | **Missing outcome data** | **Measurement of the outcome** | **Selection of the reported result** | **Overall** |
| --- | --- | --- | --- | --- | --- | --- |
| HOST-EXAM (2021) | Low risk | Some concerns | Low risk | Low risk | Low risk | **Some concerns** |

**Supplementary Table S8**: Summary of the Newcastle-Ottawa scale quality assessment for non-randomized studies

| **Study** | **Selection** | **Comparability** | **Exposure or Outcome** | **Final score** |
| --- | --- | --- | --- | --- |
| Park et al (2016) | ☆☆☆☆ | ☆☆ | ☆☆☆ | **9** |
| Sim et al (2020) | ☆☆☆☆ | ☆☆ | ☆☆ | **8** |
